# Associations between Neighborhood Open Space Features and Walking and Social Interaction in Older Adults—A Mixed Methods Study

**DOI:** 10.3390/geriatrics4030041

**Published:** 2019-07-06

**Authors:** Tanja Schmidt, Jacqueline Kerr, Jasper Schipperijn

**Affiliations:** 1 Research Unit for Active Living, Department of Sports Science and Clinical Biomechanics, University of Southern Denmark, Campusvej 55, 5230 Odense M, Denmark; 2 Department of Family Medicine and Public Health, University of California, San Diego, 9500 Gilman Dr, La Jolla, CA 92093, USA

**Keywords:** older adults, neighborhood open space, walking, social interaction

## Abstract

Neighborhood Open Spaces (NOS) such as public spaces around people’s homes, parks and village greens, may support activity and socializing for older adults. These spaces might be especially important for older adults as they typically are less mobile and have smaller activity spaces and social networks than other age groups. The present exploratory sequential mixed methods study investigates the association between built environment features, social interaction, and walking within NOS, among older adults living in a low socio-economic neighborhood in Copenhagen. Interviews, the Community Park Audit Tool, and the System for Observing Play and Recreation in Communities (SOPARC) were used to capture quantitative and qualitative data on 353 older adults (59–90 years old) within 11 NOS. Walking was predicted by the condition and shade along paths, seating and landscaping. Social interaction was negatively associated with walking, suggesting that older adults tend to sit down when engaging in social activities. Interviews highlighted the importance of social interaction within NOS. Future designs of NOS should acknowledge the importance of social meeting places, but at the same time provide walkable spaces for older adults to promote healthy aging.

## 1. Introduction

Older adults (60+) are a rapidly growing population group in most industrialized countries. They are expected to increase to 22% of the worldwide population by 2050, resulting in more older adults than children [1]. This increase will lead to major financial burdens on governments. Health care costs usually increase with age due to greater health problems, physical impairments and mental health problems [2]. Some of the most common health issues among older adults are chronic diseases related to obesity, diabetes, arthritis, hypertension, heart disease, and cancer [3], as well as depression [4] and anxiety [5]. Regular physical activity (PA), for example walking, has been shown to have numerous health benefits for older adults, such as preventing cardiovascular disease, hypertension, diabetes, depression, anxiety, cancer, high cholesterol and blood pressure, obesity, and risk of falls [6,7,8]. For older adults, walking within their community may be a safe and easy way to meet PA guidelines. However, worldwide almost a third of 60–79 year olds and half of 80+ year olds, do not meet the recommended PA guidelines of at least 150 min of moderate-intensity aerobic PA per week [9] and over a third of 60+ year olds in Denmark do not meet the recommended levels of PA [10]. Creating spaces in communities which meet older adults’ specific needs might help decrease sedentary time, increase PA through walking, and improve health [11,12].

Disparities in health and environments by socioeconomic status (SES), especially in older adults, suggest that understanding specific predictors in low-income neighborhoods would be beneficial. Low SES older adults are less active with poorer health then older adults with a higher SES [13,14,15]. Low-SES neighborhoods also provide fewer activity-friendly public open spaces and are less walkable than high-SES neighborhoods [13,16,17]. Studies of parks have reported less frequent use, poorer perceived accessibility and safety, and poor perceived distance to public parks in more deprived areas [18,19]. Given the impacts of environment and activity on health and healthcare costs, understanding the perceived barriers and facilitators for walking within low-income communities and how specific built environmental features may promote walking should thus be a key concern of health promoters and urban planners. 

Neighborhood Open Spaces (NOS) are defined as public open spaces near where people live, such as public spaces around people’s homes, neighborhood parks and community gardens, as well as village greens [20]. Since NOS are close to people’s homes and often act as social meeting places [21,22], they might be especially important for older adults, who typically are less mobile and have smaller activity spaces and smaller social networks than other age groups [23,24,25]. Especially for disadvantaged neighborhoods, small-scale changes to the built environment within NOS may be particularly beneficial, as they are easier and less costly than having to make improvements on larger scales e.g., the walkability of the whole neighborhood. Studies of low-income NOS are needed.

Several studies have investigated the association between built environment characteristics and walking, mobility or physical activity in older adults [26,27,28,29,30,31] and found safe, walkable, green and aesthetically pleasing neighborhoods with access to different destinations and services to be important. However, these studies have focused on the overall characteristics of neighborhood built environments rather than specific attributes within NOS associated with walking. Studies on attributes of NOS and their association with walking within these NOS for older adults are limited. One study by Sugiyama et al. [32] used self-reported data on older adults’ time spent outdoors walking and quality of life, and its association with self-reported quality of NOS, and found safety and pleasantness in NOS to be relevant for older adults’ life satisfaction, as well as the quality of paths to open spaces to be associated with total walking. Another study by Sugiyama and Thompson [33], conducted in the United Kingdoms (UK), found that the quality of paths to NOS, lack of nuisance, attractiveness, and facilities within the NOS (e.g., toilets) were associated with total recreational and transport-related walking. Lastly, a study by Aspinall et al. [20] found similar features like nuisances, attractions, as well as trees and heavy traffic to be of relevance for older people’s park preference—not specifically walking. Studies not focusing specifically on NOS but on gardens or parks, also found safety concerns, as well as accessibility and walkability to be associated with walking [18,34]. Although studies presented similar results, they also had similar limitations. First, most of the studies were conducted in the UK, leaving the question of whether these attributes are relevant in other European countries. Second, they used self-reported data on walking or built environmental attributes. Third, they did not investigate walking within NOS but rather walking or physical activity in general. Studies with objective measures of walking within NOS are needed.

Having a built environment that supports walking may also affect social interaction and social support among older adults. One study by Richard et al. [35] found that having a local environment that was unsupportive for walking might decrease social interaction, which consequently might lead to a decline in physical functioning due to less activity and an increase in isolation due to less social support [36]. However, living in more walkable neighborhoods supporting social interactions and having built environment features that facilitates social interaction like porches, was related to fewer depressive symptoms, less anxiety and higher quality of life [37,38,39]. A few studies have investigated the relationship between green spaces within neighborhoods and social interaction for older adults, and found that social interaction is influenced by the availability of trees, grass and greenness of the green space, along with safety and maintenance [40,41,42]. While social interaction is important for older adults, it is not clear whether features that support social interaction might detract from walking [32]. Older adults often have cognitive and cardiovascular challenges to walking and talking. However, research suggests that older adults sometimes develop highly fixed walking routines to maintain well-being, and social interaction might be part of these routines [43,44,45]. As routines usually do not take much effort, it may be possible to engage in some social interaction like talking, while walking. It is therefore important to explore social interaction as a mediator of the relationship between NOS attributes and walking.

A key issue that should concern urban planners, architects and landscape designers is how to design or reshape existing NOS to improve physical and social health in older adults with low SES. The limited amount of research in this area may have resulted in NOS which do not seem suitable or favorable for active living by disadvantaged older adults. Consequently, in order to inform improved designs of NOS for older adults, it is necessary to increase our knowledge on what specific NOS features are important for their walking behavior and how social interaction may also arise. Previous studies have relied on self-reported data, which consequently may have biased their results. However, as older adults are a quite heterogeneous group (e.g., differences in physical abilities, mental disorders, socio-economic status), and older adults living in disadvantaged neighborhoods are found to have more negative perceptions of their neighborhood [18], researchers should also not rely solely on objective measurements. To incorporate both views, researchers have proposed the use of triangulation [32]. A mixed-methods approach, applying qualitative and quantitative measurements, may improve the evidence base for designing NOS and may provide a more comprehensive understanding.

To fill this gap in the literature, the aim of this study was to identify specific features within NOS, like greenery and facilities, associated with older adults’ objectively measured walking behavior within the NOS, and to assess the association between social interaction and older adults’ walking behavior in a deprived neighborhood of Copenhagen. This was done using a sequential exploratory mixed method design with equal emphasis on QUANT and QUAL. First, we analyzed findings from several interviews conducted with older adults and identified perceived key features important for NOS use. This initial qualitative step was used to identify and adapt the most suitable auditing tool to capture those features in the NOS. Second, we quantitatively assessed the association between the identified features within the NOS, social interaction and walking among older adults and discussed the results in relation to the qualitative findings. 

## 2. Materials and Methods

### 2.1. Design and Setting

This study is part of a larger quasi-experimental intervention study (Move The Neighbourhood Study), which builds on principles from Community-Based Participatory Research and uses co-design approaches to develop tailored interventions within NOS in senior-housing areas to improve physical activity and social interaction for older adults living in a deprived neighborhood of Copenhagen, Denmark (DK) [46]. The quantitative and qualitative data collected in this larger intervention study are used for this paper to specifically investigate NOS walking behavior. 

**Figure 1:**
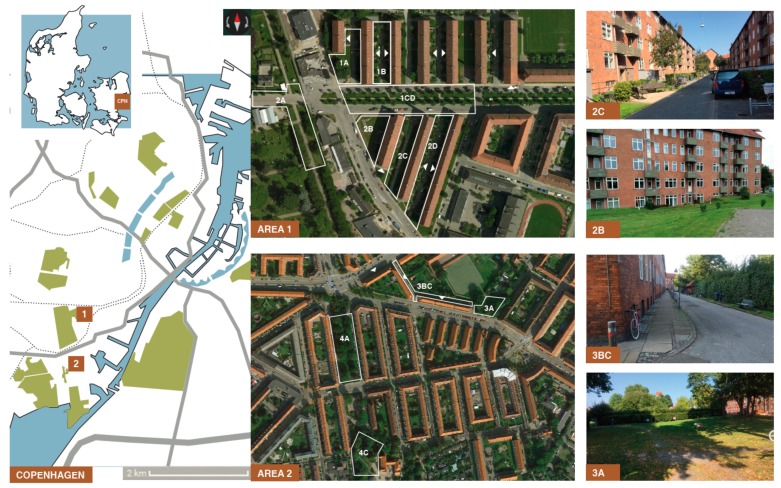
Map of Copenhagen, Sydhavnen and all 11 Neighborhood open spaces within the two senior housing areas. The map (on the left) includes information on parks (green), water (blue), major roads (grey lines), and the train system (dotted lines). White arrows show the entrances to the buildings.

The study took place in one of Copenhagen’s most disadvantaged neighborhoods, as 32.0% has no formal education (21.3% on average in DK) and 40.2% has a low income (30.6% on average in DK), with a life expectancy of 73.0 years; one of the lowest in Denmark and well below the average of 80.6 years [47]. The area is called Sydhavnen (South Harbor), and has 10.276 inhabitants [48] within a 1.2 km^2^ area, framed by high-traffic corridors. This neighborhood consists of several different types of housings, for all age groups and needs. There are several senior housing areas with apartment blocks designed for seniors’ needs, including public open spaces in and around the apartment blocks. Out of the three existing and invited housing associations within Sydhavnen containing senior housing, two of them agreed on participating in the study. The land use surrounding the first housing area consists primarily of residential buildings, a school, a pub, and a large cemetery that is also used as a recreation area. The second housing area is situated in a more urban context surrounded by mixed land use including residential buildings, shops, cafés, pubs, community centers, a school, and a town square. Eleven NOS surrounding the two senior housing areas were selected to be part of the data collection based on the following criteria: (1) they had to be next to or close to (max 400 m walking distance) one of the two senior housing areas; (2) they had to be public open spaces accessible by the target population and the general public; and (3) they had to be large enough for people to be able to stay in the NOS and do activities (e.g., small grassy spots were excluded). Figure 1 depicts all 11 NOS within the two senior housing areas. 

### 2.2. Ethics

The study and its data-management procedures were approved by the Danish Data Protection Agency (2015–57-0008). All participants signed a consent form agreeing to take part in the study. In order to ensure the participants’ anonymity, names were changed when analyzing and drafting this paper. Participants could withdraw from the study at any time. Public observations of NOS behavior did not require consent as no identifiable information was collected on participants. 

### 2.3. Mixed-Methods Approach

A mixed-methods approach, called an exploratory sequential mixed methods design [49], was used to investigate the specific design features within NOS on older adults’ walking behavior and its association with social interaction. The first purpose of the exploratory sequential mixed-methods design was to adapt and apply a quantitative measurement instrument to the specific population being investigated. This approach leads to a culture or setting-specific quantitative instrument which reflects the specific target population or setting being investigated [49]. This approach makes it possible to investigate the association between NOS features and walking for a particular study population, older adults, with specific needs, physical and mental limitations and other barriers, which are not common for other age groups. Using a measurement instrument that is not adapted to this specific target population could lead to unclear results and consequently, poor recommendations for NOS design for older adults. The second purpose of this mixed methods design was to further qualify the quantitative results by including qualitative data from the specific population being studied in the discussion of the results. 

**Figure 2:**
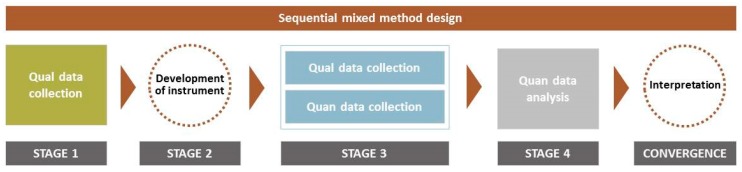
Mixed Methods diagram explaining the design and structure of this study. Qual = qualitative, Quan = quantitative.

The design and structure of the sequential mixed methods approach used in this study is explained in Figure 2. The mixed methods approach is divided into four stages. In the first stage (the exploratory stage), home-administered structured interviews were carried out in the fall of 2016 to assess the experiences of local older adults with outdoor open spaces, regarding barriers and facilitators to use them. 

In the second stage (the development stage), a validated quantitative instrument called the Community Park Audit Tool (CPAT) [50] was used and adapted based on the themes that emerged from the results of the structured interviews. In the third stage, quantitative data were collected using the adapted CPAT instrument as well as the System for Observing Play and Recreation in Communities (SOPARC) [51] in both the fall of 2016 and spring of 2017. These data were analyzed using Binomial Logistic Regression analyses to explore associations between specific NOS features, social interactions and walking in older adults. In the fourth stage, semi-structured home interviews were carried out in spring 2018 to explore older adults’ use or none-use of NOS that might support or contradict the quantitative findings. Finally, integrated conclusions were drawn based on a compilation of the qualitative and quantitative results, using stories and quotes from the qualitative interviews. The individual steps are described in details in the following. 

## 3. Stage 1—Exploration

### 3.1. Sampling

For the exploratory stage, seniors living in the two pre-identified senior housing areas were invited to participate in the home-administered structured interview. Recruitment took place during social activities hosted by the housing areas. Everyone aged 60 years and above could participate no matter their physical ability and potential impairment. Both verbally and in writing, participants were invited to be visited at home for the interviews or to meet the researcher at their local office. 

### 3.2. Procedure 

The aim of this exploratory investigation was to gain knowledge about older adults’ perceived and experienced barriers and motivators for using the local environment. Structured home-administered interviews were carried out to assess attitudes towards specific built environmental features identified in the literature to be important for older adults to use their local neighborhood. During August and September 2016, 34 face-to-face interviews were carried out. Respondents were asked if they experienced a range of built environmental barriers in their neighborhood like not enough green space, lack of seating options, safety issues, and quality of paths. Participants were also able to add any barriers not covered by the interviewer in an open-ended question. Additional demographic information was recorded and the interview took 20–30 min. The answers were written down by the interviewer. 

### 3.3. Analysis

To analyze the data from the first structured home interviews, a deductive thematic approach [52] was used to code the open-ended questions into categories of different built environmental features not covered by the initial structured interview questions. The coded categories were together with the structured interview categories combined in a matrix to identify how many respondents comment on the different categories to identify level of importance. 

### 3.4. Results

The results from the first home interviews were used to qualify the CPAT instrument. Categories not found to be important for the respondents were not included in the CPAT tool, whereas additional categories which many of the respondents mentioned as important were included in the CPAT tool, if they were not already present. The mean age of the respondents was 74, 73.5% were female and 70.6% lived alone. Categories found to be the most important for the respondents were: weather condition, condition of paths (uneven surfaces, curb cuts, to narrow paths for wheelchair users), lack of seating, and safety concerns (large groups of people, traffic, crime and lighting). Categories found to be least inadequate were green spaces, as well as cycle paths and pedestrian paths (please see the supplementary Table S1 for more information on the results). 

## 4. Stage 2—Adaption of the CPAT Instrument

### Procedure

CPAT was preliminarily chosen because of its good content validity and reliability in mapping of community parks [50]. CPAT is a user-friendly auditing tool that allows reliable auditing in different community parks and other green spaces and assesses their potential to promote physical activity. While there are other tools relevant for older adults, most do not consider perspectives of low-income neighborhoods and physical activity, which is why CPAT was chosen. It consists of four sections including several questions about access and surrounding neighborhoods, park activities, and park quality and safety. The original full version of CPAT can be found on the Active Living Research website [53]. 

During stage 2, CPAT was adapted for the specific target population and purpose of the study, by identifying similarities and differences between CPAT variables and stage 1 identified barriers and motivators (see the modified tool in the supplementary File S1). Built environment features that were identified as important during stage 1, but not part of CPAT, were added to the auditing tool, and items in CPAT that did not fit the specific context, were removed or revised. Elements excluded from CPAT were those in ‘Section 3: Park Activity Areas’ related to specific activity areas not found in NOS, like a sport field, basketball court and skate park. Questions about general activity areas like ‘trail’ and ‘green space’ were kept in the instrument. 

## 5. Stage 3—Exploration

### 5.1. Sampling

For the second exploratory stage, the same seniors living in the two pre-identified senior housing areas and who participated in the first interview were invited to participate in the semi-structured home interview. Both verbally and in writing, participants were invited to be visited at home for the interviews or to meet the researcher at their local office. 

### 5.2. Procedure 

The aim of this second exploratory investigation was to gain knowledge about older adults’ use of NOS, their reasons for using or not using the different NOS, their qualities and challenges. Using semi-structured interviews allowed for guiding the conversions on specific topics, but at the same time allowed in-depth questions about topics that may or may not be covered by the interview-guide. During spring 2018, 10 face-to face semi-structured home interviews were carried out. Topics covered in the interview were their use or none-use of their NOS, what they liked or did not like, and social interaction. Mainly, the interviews had the form of a natural conversation and questions were asked based on the elements in the neighborhood, which prompted reactions by the participants. A mobile phone was used for voice recording after asking for the respondent’s permission to use it. 

### 5.3. Analysis

To analyze the semi-structured interviews, the same deductive thematic approach [52] was chosen as for the first home-interviews, which allowed for the specific CPAT categories to direct the findings. The researcher first transcribed the audio-recorded interviews, and afterwards created themes that matched the CPAT categories. The transcripts were then coded into these CPAT themes. Lastly, a thematic analysis was conducted to create a detailed description and understanding of the transcripts. Using triangulation, by comparing quantitative data with qualitative data enhanced the understanding and rigor. The transcription software called NVIVO [54] was used to transcribe and code the interviews. 

### 5.4. Results

The following table (Table 1) highlights the main characteristics of the participants in the second interviews. In total, 10 older adults (69–89 years old, mean age 78.7, 33.3% men) participated in the interview. None of the participants worked anymore. Years of living in the neighborhood span from 3.5–25 years highlighting the variety of interviewed participants. Four out of 10 participants used a mobility aid allowing the researcher to study multiple ways of moving around the neighborhood. The majority of the participants lived alone which may influence the importance of social relations outside their individual flat [55]. 

The thematic categories identified as the most important by the interviewed older adults based on CPAT variables, in the following order, were: seating (benches or picnic tables), social interaction, landscaping (trees, bushes, and flower beds), shelter, wheelchair friendliness, and shade. The specific barriers and motivators for each identified theme, including quotes, are presented in the additional file together with the CPAT variables (Please see the supplementary Table S2). Categories identified as important were included in the quantitative analysis and further used in the discussion of the quantitative results. 

## 6. Stage 3—Quantitative Observation 

### 6.1. Measures

The 11 NOS surrounding the two senior housing areas were used to collect quantitative data in stage 3. Users of all 11 NOS were observed using SOPARC, and each of the 11 NOS’ built environment features were mapped using CPAT, chosen based on the preliminary qualitative analysis.

#### 6.1.1. SOPARC: behavioral observation tool

SOPARC was employed to observe walking behavior (dependent variable) and social interaction (independent variable) within the preselected NOS for all age groups. SOPARC is a validated and reliable tool [51] which has been used in many studies for several years to record the use of mostly parks and urban green spaces [56], but can be used for NOS as well. The original version of SOPARC was created by Thomas McKenzie and colleagues in 2006 [51], and it was modified to fit the purpose of this study. Information about age, gender, social interaction (i.e., two or more persons talking, walking, running biking, and sitting together) and primary activity (e.g., walking, sitting and talking, and biking), as well as weather conditions, lighting, and the time of observation was captured by trained researchers using the tool pictured in the additional electronic file (Please see the supplementary Figure S1). 

Based on an earlier study by Cohen and colleagues [57], we decided that 4 days of observations (3 weekdays and 1 weekend day) with four observations each day (morning, lunch, afternoon, evening) in each of the 11 NOS, would be sufficient to capture the use of those NOS. To ensure high variability in observations, the same NOS was never observed twice on the same day, and all observations were conducted twice over a 1½ month period during the fall of 2016 and spring of 2017. 

#### 6.1.2. CPAT: Environmental Observation Tool

As the CPAT tool was adapted based on findings from stage 1, which was carried out in the fall of 2016, the tool was only used during the second data collection conducted in spring 2017. There was no indication that the 11 NOS had changed between the fall of 2016 and spring of 2017 and weather conditions in Denmark are similar during the spring and fall. During the spring of 2017 data collection, a trained researcher visited all 11 NOS with a paper version of the adapted CPAT and audited each NOS accordingly. The CPAT data were digitized and matched with the SOPARC observations for each of the 11 NOS. 

## 7. Stage 4—Quantitative Analysis 

### 7.1. Analysis 

The analysis of the qualitative interviews identified different built environment attributes along with social interaction as important for the participants, in order to use their NOS for various activities (see Table S1 in online supplementary material). Based on an integrated analytical approach, these qualitative data were used to inform the quantitative analysis by guiding decisions about the inclusion of variables in the analysis. For the quantitative analysis, Binomial Logistic Regression analysis was employed using IBM SPSS Statistics 24. Logistic regression can be used to understand whether an outcome, in this case walking behavior, can be predicted based on a range of independent variables, in this case built environmental features and social interaction. Based on the relatively small sample size (n = 353), we were not able to include all variables identified by the older adults to be important. Thus, we chose to include those variables in the model that the qualitative analysis identified to be important by the majority of the interviewed population. 

The following variables collected by the CPAT instrument were included in the regression model: benches (the number of benches within a NOS), picnic tables (the number of picnic tables within a NOS), landscaping (flower beds, pruned bushes), green space shade (whether there is shade within a green space), path shade (whether there is shade on a walking path), path wheelchair friendly (is a wheelchair able to pass, get on/off the path, wide enough path), and path conditions (whether the path conditions are good or bad based on the presence of holes and uneven surface). To test the importance of social interaction identified in the qualitative analysis, the variable *social interaction* (if two or more people were observed to talk, walk or sit together, they were considered to interact socially with each other) was included in the model as a binary variable (0 = no social interaction, 1 = social interaction) as an independent variable. The outcome variable walking was also coded as a binary variable based on the SOPARC observations (0 = no walking, 1 = walking). 

Covariates included in the model were age (60+), gender and NOS size (size of each NOS in square meters). The size of each NOS was included in the model as this might affect the number of people visiting the NOS and the availability of different built environment features. 

### 7.2. Results—Association between Observed Walking, Social Interaction and CPAT Features

A total of 353 older adults were observed in the 11 NOS during data collection. Descriptive statistics are presented in Table 2. Mean age is quite low (66.73) indicating that mostly younger older adults were observed. A high percentage of older adults are walking within the NOS (72%), which, not surprisingly, matches the relatively low percentage of older adults engaging in social interaction (30%). Gender is evenly distributed (48.2% females). Forty-five percent of older adults were observed in NOS that had landscaping, and an average of 5.37 benches was registered, whereas less than on average 1 picnic table was registered in all the NOS. 

Table 3 represents results from the Binomial Logistic Regression analysis on *walking.* The model is significant (*p* = 0.000) and predicts 78.5% of all cases correctly. Odds ratio, 95% confidence intervals, crude rate for participants walking (crude %), and significance levels of each variable are presented in the table, and variables with acceptable *p*-values (<0.05) are highlighted. Age, landscape, picnic tables, path (shade), path (condition), and social interaction are all significantly associated with walking in older adults, some negatively and some positively. Social interaction seems to have a negative impact on walking (odds ratio = 0.223), as 80.2% walk alone, whereas only 52.8% walk while engaging in social interaction. This indicates that older adults tend to be sedentary when socializing. This might also explain why benches are not significantly associated with walking (*p* = 0.223). Shady paths was also negatively associated with walking (odds ratio = 0.023), as older adults were less likely to walk if shade was present. This suggests that they rather want to walk in sunny places; however, shady green space was not significantly associated with walking. The odds of walking within NOS is greatly affected by the condition of walking paths (odds ratio = 9.695), suggesting that well-maintained paths are important for older adults to walk. Landscaping (odds ratio = 0.303) was surprisingly negatively associated with walking, as one might think that the presence of flower beds and bushes have a positive impact on the odds of walking. The odds of walking were 1.569 times higher for each additional picnic table in the NOS, suggesting that the presence of some kind of seating is important for walking. Lastly, the odds of walking were significantly affected by age. The covariates’ gender and NOS size were not significantly associated with walking. 

## 8. Discussion

The aim of this mixed-methods study was to investigate the association between walking behavior within NOS and different built environmental features of NOS, and how social interaction might affect this behavior.

To our knowledge, this is the first study using a mixed-methods approach to investigate built environment characteristics within NOS and its association with older adults’ walking behavior and social interaction in NOS, rather than general walking behavior or walking to and from NOS as seen in previous studies. By using a mixed-methods research design, an adapted quantitative tool to assess built environment features within NOS, and afterwards qualify these findings using qualitative data, we were able to consider the specific population in the assessment of NOS rather than relying on standardized tools. During stage 1, several built environment features were identified to be important for the local older adults like seating and path conditions. Additionally, by combining qualitative and quantitative results, we were able to analyze qualitative identified built environment features in a quantitative statistical model and discuss these quantitative results using qualitative themes and quotes, which strengthened the analysis and interpretation of results. 

The results from the regression analysis indicate that built environmental features found to be associated with walking within NOS were shady paths and their condition, the presence of picnic tables, and landscaping like bushes and flowerbeds. Social interaction was also found to be highly significantly associated with walking; when social interaction was taking place, walking was less likely to occur. 

Older adults can be more fragile and might need to use assistive devices like a cane or a walker for active transportation [58]. Thus, the condition of paths within NOS may be especially important for this age group, as uneven surfaces or holes on the paths can make it difficult for older adults to navigate. This might also explain the positive association found between picnic tables and walking. As older adults are less mobile, having the option of sitting and taking a rest while walking around may be important for older adults. However, benches within NOS were not associated with walking. The study by Aspinall and colleagues on preferences of specific NOS attributes also found seating to be of importance [20], although it was only the ninth most important out of 15 attributes. However, this might be because study did not look specifically at walking in older adults. Another study found seating to be important for older adults’ walking behavior within the neighborhood [59]. Another reason could be that there is usually more activity in places that include seating, as they attract different groups of people to sit and talk, rest, read a book, and have a picnic. This might be especially attractive for older adults, as they can be socially isolated, thus, walking in a neighborhood with open space with lots of people and activity may be more interesting for older adults, as they may feel safer, which previously has been found to be important [32], less alone and more entertained with the possibility of talking to other community members [60]. As one resident mentioned; “*But I greet them all, because now I know them… they wave at me…and we exchange some words…”.*

Our results indicate that shady paths have a negative influence on walking, which contradicts previous research on shades’ association with walking [61,62]. However, both studies were conducted in Australia where summer temperatures can be really hot. Denmark has quite mild summers and dark winters, which might explain why too much shade within NOS has a negative effect on walking, as people want to enjoy the sun when it is finally shining. Additionally, data were collected during spring and fall where temperatures are quite mild. To summarize, well-maintained and sunny walking paths and the possibility of seating seem to promote walking within NOS. These physical features are relatively small and easy to implement, which is an advantage in low SES neighborhoods were funds for renovations may be limited. Contradictory, previous studies investigating different built environmental features and its association with walking in older adults, recommend rather extensive and costly neighborhood changes like improving the street connectivity and greater land-use mix [13,26,63,64]. Making small-scale changes in the local neighborhood environment may be as effective as large-scale improvements and easier to implement in smaller communities. 

Social interaction was found to be negatively associated with walking within NOS, indicating that older adults are potentially less active when interacting socially. This suggests that they may tend to sit down when engaging in social activities within NOS, rather than walking or playing together like other age groups tend to do [65,66]. This might also explain why benches were not associated with walking. By analyzing the qualitative data (found in the additional electronic file), it became clear that especially the social relationships or casual encounters around seating places with different neighbors were important for the residents. As one interviewed woman said: ”*We just sit and chat, just for a couple of hours or three and then we leave again…I have brought coffee with me and we just sit and enjoy…I really don’t want to sit there all by myself*”. This person talked about a paved space area with benches, trees and flower beds just outside her apartment building, were people usually hang out and make small talk with passing neighbors. Thus, these places seem to be for social encounters rather than walking, which may also explain the negative association found between landscaping and walking. Several interviewed people talked about these meeting places just outside their apartment buildings as a place they cherished. As most of the interviewed older adults lived alone, these social outdoor spaces in their immediate surrounding seem to be especially important for them. As one interviewed man put it: *“…well a lot of people are alone right…but then they meet down there (by the benches and raised beds) and talk…”* This quote highlights the importance of outdoor social spaces in order for older adults to maintain social engagement and counteract the loneliness that is often associated with aging. The results suggest that social interaction occurs while seating, which may be because it is easier for older adults to engage in social activities while sitting. However, other studies found that older adults do interact during routine walking as it mentally might takes less effort leaving room for social encounters [43,44,45]. The different findings might suggest that the behavior observed in this paper may be related to this specific study population of older adults living in disadvantaged communities, which were characterized by low socio-economic status and great disabilities, both mentally and physically. 

The importance of social interaction for older adults is highlighted in several studies [34,40,41,67,68]. The study by Finlay et al. discusses the importance of social green spaces particularly for people who live alone as they are at greater risk of isolation and loneliness; and Milligan et al. points out the importance of communal gardening to combat social isolation and a supportive community environment helping less able members of the group. Consequently, older adults from low socio-economic neighborhoods who live alone and have various physical challenges like the current study population may especially benefit from NOS. Hence, even though social interaction might not occur with walking in older adults within NOS, it seems to be a vital part for the elderly’s quality of life, as their social gatherings in the NOS give them a reason to get out of their apartments, which on the other hand makes them walk, as they have to leave the apartment to meet friends and neighbors in NOS. If so, walking may not occur extensively within the NOS, but on the way to the NOS, and NOS as such, are more important for social interaction than for walking. As many older adults face loneliness due to loss of their partner and friends, social relations may be especially important for this age group. One study by Yung et al. [69], investigating older adults living in urban renewal districts in Hong Kong, stressed that older adults consider social spaces and activities as their most important needs, rather than walkable and safe open spaces. This is further confirmed in another study by Yung et al. [70], who stressed the need to focus more on social spaces when planning and designing public parks, and to include the population in decision making. Consequently, it may be more important to focus on improving NOS which support social interactions for older adults rather than focusing on walkable NOS alone. By creating spaces in their immediate surroundings which support social activities or random interactions, it might be possible to decrease loneliness and increase walking to and from these NOS. However, this has to be further tested. Since many NOS already seem to be social meeting places, landscape designers and architects might focus on improving the walkability within already existing social NOS to promote walking behavior. This dilemma of where to focus your money on—walkability or social interaction—highlights the importance of using mixed methods designs, as researchers, landscape designers and decision makers need to include local older residents to pinpoint the specific needs of the local community, whether it is social meeting places or walkable places. 

### Methodological Considerations 

The strength of this study was the use of mixed methods to adapt the most appropriate mapping tool for the specific population and low SES environment being investigated and using an integrated analytical approach in the analysis and interpretation of the results. Further, previous studies on NOS and walking in older adults have relied on self-reported data which might be biased by participants’ perceptions or social desirability. The use of systematic observations and structured audits in this study is a strength, especially in combination with qualitative data to confirm findings of this heterogeneous group. 

Several limitations of the study should be considered. Using cross-sectional data limits our ability to present any causal relationships between the outcome ‘walking’ and the different demographic and built environment variables. Second, as the data were collected within a disadvantaged area of Copenhagen, it is uncertain whether the results are generalizable to other Danish or international more advantaged or disadvantaged communities. Nonetheless, the results are important in supporting the relevance of social and walkable NOS within deprived neighborhoods in a Danish and European context, which is in most need of supportive neighborhood built environments, due to their low life-expectancy, and increased health-related problems. Third, other observational tools may have been more suitable for investigating NOS in older adults, as CPAT was not specifically created for this purpose. This might explain why many of the included variables in the quantitative analysis were not significant. Fourth, even though SOPARC is a validated and reliable tool when used by trained researchers, basic skills like judgement of age can be difficult. However, since the qualitative interviews indicated the same results as the results from the regression analysis, misjudgment in age probably did not occur. Additionally, SOPARC observations were only done within the NOS and as such, information about walking to the NOS was missing, which may contribute to total daily walking. Other observational methods like go-along interviews may have provided more insight into walking routines and social encounters. Mobile assessments of walking in seniors through accelerometers and GPS might elucidate how much activity occurs on the way to NOS as well as within. Fifth, data were collected during the spring and fall, thus it is uncertain if our findings would be similar if data were collected during winter or summer. 

## 9. Conclusions

This study adds to the accumulated knowledge on how to use mixed methods to qualify measurement tools and analysis for specific target populations. Given the diversity of older adults’ health, functional status and lifestyle—especially in low SES neighborhoods—this target population may have very different attitudes and preferences for qualities within NOS, highlighting the need for using context-specific and highly tailored measurement tools in feature studies. The study further adds to the limited knowledge on how to improve NOS in disadvantaged neighborhoods for older adults, to encourage walking in the Danish (and European) context which is more densely populated and less car-dependent than in the US. NOS may be easy and affordable public spaces to promote walking in older adults by ensuring well-maintained walking paths with some shade and access to seating. These relatively affordable and small changes can easily be introduced by the municipality in disadvantaged neighborhoods and hereby promote walking and lower costs for large-scale community renovations. In contrast, other studies have focused on large neighborhood-wide and expensive changes like street connectivity and greater land use mix, which may not be feasible in low-income neighborhoods. Results from this study suggest that much social interaction occurs in NOS but this may promote sedentary behavior rather than walking. However, walking may still occur as people have to walk to and from the NOS, which will generate some level of walking. Since these NOS seem to act as social spaces rather than spaces for walking activities, landscape designers, architects and urban planners should acknowledge the importance of designing or improving NOS for older adults focusing on both social and physical health, by promoting walking to and from the NOS and simultaneously social interaction within the space. More intervention studies are needed to investigate the longitudinal effects of changing older adults’ NOS to fully understand how to design such public open spaces in order to improve both physical and social health. Future studies should assess walking to and from NOS as well as walking within NOS in combination with social interaction, to investigate how NOS promote walking and social interaction. 

## Figures and Tables

**Table 1:** Demographics of participants from the second interviews.

Housing Area	Gender	Age	Mobility Aid	Living Situation	Years of Living in Sydhavnen
1	Female	75	None	Alone	6
1	Female	74	Mobility scooter	With cousin	7
1	Female	85	Mobility scooter and walker	Alone	3.5
1	Male	78	None	Alone	11
1	Female	89	Walker	Alone	5
2	Female	74	None	Alone	4.5
2	Female	83	None	Alone	25
2	Female	69	None	Alone	4
2	Male	82	Mobility scooter and walker	Alone	5
2	Male	78	None	Alone	4

**Table 2:** Descriptive of data from SOPARC observations and CPAT registrations.

Variable	Mean	%	Range
Walking (yes)		72	
Social Interaction (yes)		30	
Gender (female)		48.2	
Age (count)	66.73		60–90
NOS size (square meters)	10,148.7920		2622.85–19,659.36
Bench (count)	5.37		0–10
Picnic table (count)	0.57		0–4
Landscape (flower beds, pruned bushes) (yes)		45	
Green space (shade) (yes)		46.5	
Path (shade) (yes)		64.9	
Path (condition) (Good)		65.4	
Path (wheelchair friendly) (yes)		57.2	

Note: NOS = Neighborhood open spaces, % = Percentage, N = 353.

**Table 3:** Binomial Logistic Regression analysis on walking (dependent variable).

Sig. of total model: *p* < 0.0001					
Overall percentage of cases predicted by the model = 78.5%					
Variable	N	Crude % or Mean	OR	95% CI for OR	Sig.
**Age** (count)	353	67	1.047	1.000, 1.095	**0.046**
**Gender**					
Female	170	72.9%	1.353	0.799, 2.290	0.261
Male	183	71.0%	1		
**NOS size** (square meters)	353	10,574.36	1.000	1.000, 1.000	0.987
**Landscape**					
Bushes, flower beds	159	68.6%	0.303	0.104, 0.882	**0.029**
None	194	74.7%	1		
**Bench** (count)	353	5	0.892	0.760, 1.045	0.157
**Picnic table** (count)	353	1	1.569	1.092, 2.255	**0.015**
**Green space shade**					
Shade	164	78.0%	0.768	0.146, 4.054	0.756
None	189	66.7%	1		
**Path shade**					
Shade	229	71.2%	0.023	0.002, 0.218	**0.001**
None	124	73.4%	1		
**Path condition**					
Good	231	73.6%	9.695	1.261, 74.550	**0.029**
Bad	122	68.9%	1		
**Path wheelchair friendly**					
Yes	202	72.8%	1.788	0.502, 6.366	0.370
No	151	70.9%	1		
**Social interaction**					
Social interaction	106	52.8%	0.223	0.129, 0.384	**0.000**
None	247	80.2%	1		

Note: N = total population, Mean = mean for participants walking (continuous variables), Crude % = crude rate for participants walking (categorical variables), Sig. = significance < 0.05 (in bold), 95% CI = Confidence intervals, OR = Odds Ratio, NOS = Neighborhood open spaces.

## Supplementary Materials

The following are available online at https://www.mdpi.com/2308-3417/4/3/41/s1, Figure S1: SOPARC observation scheme used in the present study, Table S1: Results from Stage 1: Built environment categories, Table S2: Match between quantitatively analyzed CPAT variables and qualitatively identified barriers/motivators for using NOS, File S1: Modified CPAT Audit Tool
