# Supplementary material for: Associations between Neighborhood Open Space Features and Walking and Social Interaction in Older Adults—A Mixed Methods Study
Source: Geriatrics (Basel). 2019 Jul 6;4(3):41. doi: 10.3390/geriatrics4030041 (PMC6787595; doi:10.3390/geriatrics4030041)
Supplement: Supplementary file 1 [file geriatrics-04-00041-s001.pdf]

ID	Gender		Age group				Activity Level			Social Interaction (ID number)		Primary Activity(note)	Note
	Male	Female	Child	Teen	Adult	Senior	Sed	Walk	Vig	None	Yes		
1													
2													
3													
4													
5													
6													
7													
8													
9													
10													
11													
12													
13													
14													
15													
16													
17													
18													
19													
20													
21													
22													
23													
24													
25													
26													
27													
28													
29													
30													

Date:__________ **Target area:**________

Sub area:_______ **Scan number:**______

Lightning:______ **Start time:**________

Weather:_______ **End time:**_________

Period: Morning Lunch
Afternoon Evening

Week day: Monday Tuesday Wednesday Thursday Friday Saturday Sunday

Primary Activity: fx transportation a-b, play, sitting and talking, dog walking

Social Interaction:Interaction between two or more people. Talking, playing, running, walking, biking together.

Sedentary: laying down, sitting, standing still

Walk: walking, yoga, easy play

Vigorous: running, rough play, football, fitness, cycling

Lightning and Weather

REMEMBER TO TAKE A PICTURE !!!!

Morning: 7:30 - 8:30 **Afternoon:** 15:30 - 16:30

Lunch: 11:30 - 12:30 **Evening:** 19:30 - 20:30

Age group: Child (0-13) Teen (14-20) Adult (21-59) Senior (60+)

Figure S1: SOPARC observation scheme

Structured interview categories	N
Bad weather	24
Uneven paths	21
Large groups of people on streets	19
Lack of seating options	16
Lack of public transportation	13
Crime	12
Traffic	11
Graffiti	9
Lack of traffic lights for pedestrians	7
Lack of safe pedestrian crossings	7
Lack of lighting during night time	5
Lack of interesting places to visit	4
Too few people on the streets	4
Lack of age-friendly public transportation (lowered entrance)	4
Lack of foot paths	3
Lack of bicycle paths	2
Lack of green spaces	2
**Quotes from open ended questions**	Themes
"Curb cuts are not wide enough and too steep to use with an electric wheelchair scooter, when driving on/off the streets"	Curb cuts conditions
"Uneven paths on tranehavevej (street name)"	Path conditions
"Bicycle paths not wide enough, so you drive over bumps constantly (electric wheelchair user). I only drive on the bicycle paths, not on the pedestrian paths, as it is too uneven (the path surface)"	Path conditions
"Lids on trash cans so birds do not through around the waste"	Cleanness/aesthetics
"Lack of striping at the (entrance to the) graveyard for the cars and bicycles. Lack of roads bumps and speed limit signs"	Traffic safety
"Lack of benches at the greave yard"	Seating
"Traffic lights at Mozarsplads (public place) are missing"	Traffic safety
"There is a lack of some hyggekroge (cozy hang out places) with flowers and so on"	Landscaping

Table S1: Results from Stage 1: Built environmental categories. N = number of interviewees who mention each variable. Some of the quotes match with the identified structured interview categories, but are not included in the number of interviewees who mention each variable (N).

CPAT variables	N	Quotes
Public transit stop	1	"…there are missing sheltered bus stops and so on out here right…"
Traffic signal	0	
Dog rules	3	"At (name of the housing area) the dogs are allowed to walk on the grass… you need of course to control your dog right…"
Curb cuts/ramps on sidewalks	1	"… there is this high edge at the pavillion and…well, that is of course not good for our wheelchair users … we need a ramp somewhere so that they can get to the pavilion"
Shade	6	"… if the sun is shining too bright, there is a parasol we can use… that's actually the place I like the most…"
Bench	8	"They sit a lot on those benches down there where the flowers are….. there are sitting quite a lot done there I think…and all of them know me right…so I stop there and talk a bit with them… that's fine…"
Wheelchair friendly	7	"… we were about to go over and into the garden, we had to walk like this (uses her walker to show that she had to lift it to get over the lawn) with our walkers, it was like there were moles, and then she got tired"
Condition	2	(asking about newly laid path in the garden) "yes yes, I think it is very good, when you have to get around in the garden, it is quite hard to 'drive' (using a walker with wheels) on the grass…"
Landscaping (pruned bushed and flower beds)	10	"… that's actually the place I like the most to hang out, down at the courtyard with flowers everywhere… I think it is quite nice… but I also think it's quite nice to sit in the rose garden…"
Lighting	1	(talking about people afraid of walking outside at night because of not enough lighting on the streets) "… I am the only one who dares to walk around outside during night time, I don't understand what they are afraid of"
Trees	3	(talking about a local grave yard) "Because it is a lovely park… with huge trees and large planes and so on, it's like walking around in an English park…"
Picnic table	5	"Well, I have alwasy walked down there and sat right… I have my own picnic table down there right…well, not my own, but I made sure that we got it…I sit there and sometimes other people come and sit with me"
Picnic shelter	7	"Yes, we like to sit there and drink our coffee with the others and chat and so on… and there is this… shelter you can pull out… so you are dry if it rains, or if the sun is too strong…that's actually the place I like the most"
Social interaction	10	"We just sit and chant for a couple of hours or three and then we go up (up to their apartment) again.. I have had coffee with me down there and we have sat there and had fun… I really don't like to sit down there all by my own… I really don't want to… but I have people to talk to over in the garden"<br><br>"I meet people I know when I am in Netto (supermarket)… and then we talk… It takes me a long time to get over to my garden (they have small community gardens near their apartment buildings), because I talk to everyone on the way right (laughs), well they want to right…"

Table S2: Match between quantitatively analysed CPAT variables and qualitatively identified barriers/motivators for using NOS. N = number of interviewees who mention each variable

“MODIFIED” COMMUNITY PARK AUDIT TOOL

Section 1: NOS Information

Target area: **Sub area:** **NOS Address/Location:**

Date (d/m/yr):

Temperature: _____ °C **Weather (circle answer):** Clear Partly Cloudy Rain/Snow

Section 2: Access and Surrounding Neighborhood

This section asks about accessing the NOS and about the neighborhood surrounding the NOS. **When thinking about the surrounding neighborhood, consider all areas that you can see from inside of the NOS.**

When rating the access and surrounding neighborhood, please use the following definition:

- **Useable**: everything necessary for use is present and nothing prevents use (e.g., sidewalks are passable)

1. Can the NOS be **accessed for use**? (e.g., not locked/fenced, available for activity, etc.)
   - No (0)
   - Yes (2)
   - Only by residents (1)

2. How many **points of entry** does the NOS have?
   - More than 5 (or park boundary is open) (2)
   - 2-5 (1)
   - Only 1 (0)

3. Is there a **public transit stop** within sight of the NOS?
   - No (0)
   - Yes (1)

4. What types of **parking** are available for the NOS? *(check all that are present)*
   - None (0)
   - Parking Lot (1)
   - On street parking (1)

- Bike rack(s) (1)

5. Are there **sidewalks** on *any* roads bordering the NOS? (could be on opposite side of road)
    - No (0)
    - Yes (1)

    a. If yes … Are they useable?
        - All or most are useable (3)
        - About half (2)
        - None or few useable (0)
        - Not by wheelchair users (1)

    b. If yes … Are there **curb cuts and/or ramps** on *any* sidewalks bordering or entering the NOS?
        - No (0)
        - Yes (1)

6. Is there an external **trail or path** connected to the NOS?
    - No (0)
    - Yes (1)

    a. If yes … Is it useable?
        - No (0)
        - Yes (2)
        - Not by wheelchair users (1)

7. Are there **bike routes** on *any* roads bordering the NOS? *(check all that are present)*
    - None (0)
    - Marked bike lane (1)
    - Bike route sign (1)
    - Share the road signs/markers (1)

8. Are there nearby **traffic signals** on *any* roads bordering the NOS? (e.g., crosswalk, stop light/sign)
    - No (0)
    - Yes (1)

9. What are the main **land use(s) around the NOS**? *(check all that apply)*
    - None present (0)
    - Residential (1)
    - Commercial (1)
    - Institutional (e.g., school) (1)
    - Industrial (e.g., warehouse) (1)

- Natural (1)

10. Which of the following **safety or appearance concerns** are present in the **neighborhood surrounding the NOS**? *(check all that are present in the surrounding neighborhood within sight on any side of the NOS)*

    a. Poor lighting (e.g., low or no lighting on surrounding neighborhood streets) (1)
    b. Graffiti (e.g., markings or paintings that reduce the visual quality of the area) (1)
    c. Vandalism (e.g., damaged signs, vehicles, etc.) (1)
    d. Excessive litter (e.g., noticeable amounts of trash, broken glass, etc.) (1)
    e. Heavy traffic (e.g., steady flow of vehicles) (1)
    f. Excessive noise (e.g., noticeable sounds that are unpleasant or annoying) (1)
    g. Vacant or unfavorable buildings (e.g., abandoned houses, liquor store) (1)
    h. Poorly maintained properties (e.g., overgrown grass, broken windows) (1)
    i. Lack of eyes on the street (e.g., absence of people, no houses or store fronts) (1)
    j. Evidence of threatening persons or behaviors (e.g., gangs, alcohol/drug use) (1)
    k. Other: (1)
    l. None present (0)

Section 3: NOS Activity Areas

This section asks about the activity areas in the NOS. For each activity area type:

1. First, mark the number (#) of areas that are present in the NOS (if none, write "0").
2. Then, respond to questions about **up to three** of those activity areas. If there are more than three areas for a specific activity area type, **rate the first three you come across during the audit**. If there were no activity areas of that type present, move on to the next type.

When rating the activity areas, please use the following definitions:

- **Useable**: everything necessary for use is present (excluding portable equipment - rackets, balls, etc.) and nothing prevents use (e.g., are trails passable, etc.)
- **Good condition**: looks clean and maintained (e.g., minimal rust, graffiti, broken parts; even surface; etc.)
- **Wheelchair friendly**: is a wheelchair able to pass, get on/off the path? Is the path wide enough for a wheelchair.

11. Activity Areas	Answer		
**a. Garden (# :_____)**			
Useable	No (0)	Partly (1)	Yes (2)
Good condition	No (0)	Partly (1)	Yes (2)
Wheelchair friendly	No (0)	Partly (1)	Yes (2)
Shade cover for some (25%+) of the area	No (0)	Partly (1)	Yes (2)
Benches in/surrounding area	No (0)	Partly (1)	Yes (2)

Fence around area (i.e., half or more)	No (0)	Partly (1)	Yes (2)
Separation or distance from road	No (0)	Partly (1)	Yes (2)

Comments:____

b. Residential paved space (# :_____)

Useable	No (0)	Partly (1)	Yes (2)
Good condition	No (0)	Partly (1)	Yes (2)
Wheelchair friendly	No (0)	Partly (1)	Yes (2)
Shade cover for some (25%+) of the area	No (0)	Partly (1)	Yes (2)
Benches in/surrounding area	No (0)	Partly (1)	Yes (2)
Fence around area (i.e., half or more)	No (0)	Partly (1)	Yes (2)
Separation or distance from road	No (0)	Partly (1)	Yes (2)

Comments:____

c. Residential green space (# :_____)

Useable	No (0)	Partly (1)	Yes (2)
Good condition	No (0)	Partly (1)	Yes (2)
Wheelchair friendly	No (0)	Partly (1)	Yes (2)
Shade cover for some (25%+) of the area	No (0)	Partly (1)	Yes (2)
Benches in/surrounding area	No (0)	Partly (1)	Yes (2)
Fence around area (i.e., half or more)	No (0)	Partly (1)	Yes (2)
Separation or distance from road	No (0)	Partly (1)	Yes (2)

Comments:____

d. Trail (# :_____)

Useable	No (0)	Partly (1)	Yes (2)
Good condition	No (0)	Partly (1)	Yes (2)
Wheelchair friendly	No (0)	Partly (1)	Yes (2)
Shade cover for some (25%+) of the area	No (0)	Partly (1)	Yes (2)
Connected to activity areas	No (0)	Partly (1)	Yes (2)
Benches along trail	No (0)	Partly (1)	Yes (2)
What is the trail surface? *(check one)*	Paved (3)		
	Crushed stone (2)		
	Dirt/mulch (1)		

Comments:____

e. Other (*fill in a type description):*____________________

Useable	No (0)	Partly (1)	Yes (2)

Good condition	No (0)	Partly (1)	Yes (2)
Wheelchair friendly	No (0)	Partly (1)	Yes (2)
Shade cover for some (25%+) of the area	No (0)	Partly (1)	Yes (2)
Benches in/surrounding area	No (0)	Partly (1)	Yes (2)
Fence around area (i.e., half or more)	No (0)	Partly (1)	Yes (2)
Separation or distance from road	No (0)	Partly (1)	Yes (2)

Comments: ____

Section 4: NOS Quality and Safety

This section asks about factors related to comfort and safety when using the NOS.

When rating the quality and safety features, please use the following definitions:

- **Useable**: everything necessary for use is present and nothing prevents use (e.g., can get into restrooms, drinking fountains work, etc.)
- **Good condition**: looks clean and maintained (e.g., minimal rust, graffiti, broken parts; etc.)

12. Are there public **restroom(s) or portable toilet(s)**?
    - No (0)
    - Yes (2)
    - Only for residents (1)

    a. If yes …
    Are the restroom(s) useable?
    - All or most are useable (3)
    - About half (2)
    - None or few are useable (1)
    - Don´t know (0)

    Are they in good condition?
    - All or most in good condition (3)
    - About half (2)
    - None or few in good condition (1)
    - Don´t know (0)

13. Are there **drinking fountain(s)** at the NOS?
    - No (0)
    - Yes (1)

    a. If yes …
    How many different fountains are there? (i.e., units, not spouts) ______

    Are the fountains useable?
    - All or most are useable (3)

- About half (2)
- None or few are useable (1)

Are they in good condition?
- All or most in good condition (3)
- About half (2)
- None or few in good condition (1)

14. Are there **bench(es)** to sit on in the NOS?
    - No (0)
    - Yes → amount:________ (1)

a. If yes …
   Are the benches useable?
   - All or most are useable (3)
   - About half (2)
   - None or few are useable (0)
   - Not by wheelchair users (1)

   Are they in good condition?
   - All or most in good condition (3)
   - About half (2)
   - None or few in good condition (1)

15. Are there **picnic table(s)** in the NOS?
    - No (0)
    - Yes → amount:_________ (1)

a. If yes …
   Are the tables useable?
   - All or most are useable (3)
   - About half (2)
   - None or few are useable (0)
   - Not by wheelchair users (1)

   Are they in good condition?
   - All or most in good condition (3)
   - About half (2)
   - None or few in good condition (1)

   Is there a picnic shelter in the NOS?
   - No (0)
   - Yes (1)

Is there a grill or fire pit in the NOS?

- No (0)
- Yes (1)

16. Are there **trash cans** in the NOS?
    - No (0)
    - Yes → amount:________ (1)

    a. If yes …
    Are they overflowing with trash?
        - All or most overflowing (1)
        - About half (2)
        - None or few overflowing (3)

    Are they near activity areas?
        - All or most are near (3)
        - About half (2)
        - None or few are near (1)

    Are recycling containers provided?
        - No (0)
        - Yes (1)

17. If the sun was directly overhead, how much of the NOS would be **shaded**?
    - <25% (1)
    - 25-75% (2)
    - >75% (3)

18. Are there **rules posted about animals** in the NOS? (e.g., dogs must be leashed)?
    - No (0)
    - Yes (1)

19. Is there a place to get **dog waste pick up bags** in the NOS?
    - No (0)
    - Yes (1)

    a. If yes … Are bags available at any of the locations?
        - No (0)
        - Yes (1)

20. Are there **lights** in the NOS? (not including neighborhood street lights)
    - No (0)
    - Yes (1)

a. If yes …
How much of the NOS could be lit?
- <25% (1)
- 25-75% (2)
- >75% (3)

Are the activity areas lit?
- All or most are lit (3)
- About half (2)
- None or few are lit (1)

21. From the center of the NOS, how **visible is the surrounding neighborhood**?
    - Fully (3)
    - Partially (2)
    - Not at all (1)

22. Are there **road(s)** of any type through the NOS?
    - No (1)
    - Yes (0)

    a. If yes … Are there traffic control mechanisms on the roads within the NOS? (e.g., crosswalk, stop light or sign, brick road, speed bumps, roundabouts) ☐
       - No (0)
       - Yes (1)

23. Which of the following **quality or safety concerns** are present **in the NOS**? *(check all that are present)*
    - Graffiti (e.g., markings or paintings that reduce the visual quality of the area) (1)
    - Vandalism (e.g., damaged signs, buildings, equipment, etc.) (1)
    - Excessive litter (e.g., noticeable amounts of trash, broken glass, etc.) (1)
    - Excessive animal waste (e.g., noticeable amounts of dog waste) (1)
    - Excessive noise (e.g., noticeable sounds that are unpleasant or annoying) (1)
    - Poor maintenance (e.g., overgrown grass/weeds/bushes or lack of grass in green areas) (1)
    - Evidence of threatening persons or behaviors (e.g., gangs, alcohol/drug use) (1)
    - Dangerous spots in the park (e.g., abandoned building, pit/hole) (1)
    - Other ____ (1)
    - None present (0)

24. What **aesthetic (i.e., beautiful/pleasing) features** are present **in the NOS**? *(check all that are present)*
    - Evidence of landscaping (e.g., flower beds, pruned bushes) (1)
    - Artistic feature (e.g., statue, sculpture, gazebo, fountain) (1)

- Historical or educational feature (e.g., monument, nature display, educational signs, etc.) (1)
- Wooded area (e.g., thick woods or dense trees) (1)
- Trees throughout the NOS (e.g., scattered trees) (1)
- Water feature (e.g., lake, stream, pond) (1)
- Meadow (e.g., natural, tall grassy area) (1)
- Other ____ (1)
- None present (0)

List the facilities and numbers of facilities within the NOS:

- .
- .
- .
- .
- .
- .
- .
- .
- .
- .
-
